# Virtual endocast of the Late Miocene *Hoplitomeryx matthei* (Artiodactyla, Hoplitomerycidae) and brain evolution in insular ruminants

**DOI:** 10.1098/rspb.2025.1542

**Published:** 2025-09-03

**Authors:** Pierre Orgebin, Alexandra van der Geer, George Lyras, Bastien Mennecart, Grégoire Métais, Roberto Rozzi

**Affiliations:** ^1^ Zentralmagazin Naturwissenschaftlicher Sammlungen, Martin Luther University Halle-Wittenberg, Halle (Saale), Germany; ^2^ Museum für Naturkunde – Leibniz-Institut für Evolutions- und Biodiversitätsforschung, Berlin, Germany; ^3^ Institute of Biology, Faculty of Life Sciences, Humboldt-Universität zu Berlin, Berlin, Germany; ^4^ Naturalis Biodiversity Center, Leiden, The Netherlands; ^5^ Faculty of Geology and Geoenvironment, National and Kapodistrian University of Athens, Athens, Greece; ^6^ Geosciences, Naturhistorisches Museum Basel, Basel, Switzerland; ^7^ Department of Origin and Evolution, Museum National d'Histoire Naturelle, Paris, France

**Keywords:** endocast, geometric morphometrics, Miocene, island evolution, Ruminantia

## Abstract

Mammals often follow peculiar evolutionary trajectories on islands, with some Pleistocene insular large mammals exhibiting reduced relative brain size. However, the antiquity of this phenomenon remains unclear. Here, we report the first digital endocast of an insular artiodactyl, the five-horned ruminant *Hoplitomeryx matthei* from the Late Miocene Gargano palaeo-island (Apulia, Italy). We compare its brain morphology with that of extant and extinct relatives, including the early bovid *Eotragus* and the Mid-Miocene cervid *Euprox*, and investigate endocranial size and shape variation across 35 ruminant species. *H. matthei* displays a derived pecoran brain morphology, similar to that of bovids. This finding suggests that its ancestor, rather than deriving from an Oligocene member of Tragulina, was a Pecora and colonized Gargano no earlier than the Early Miocene. This is further supported by its encephalization quotient and the presence of a prominent marginal pole at the top of its endocasts, also found in Caprini. However, unlike the Balearian mouse goat *Myotragus balearicus*, *H. matthei* does not exhibit a reduced occipital region of the neocortex or olfactory bulbs. Instead, it underwent only a minor brain size reduction, highlighting distinct pathways of brain evolution in different island ecosystems. This study provides new insights into the biogeographic history of *Hoplitomeryx* and the palaeoneuroanatomy of insular mammals prior to the Quaternary.

## 1. Background

Islands, with their inherent isolation and distinctive characteristics, offer a unique framework for investigating the processes driving evolutionary diversification [1–5]. Peculiar demographical, physiological, behavioural and morphological features have been documented in insular vertebrates and are referred to as the ‘island syndrome’ [6–9]. Among the most spectacular of these adaptations are body size changes, ranging from gigantism in small species to dwarfism in large species, as predicted by the ‘island rule’ [10–14]. Evolutionary body size reduction has been recorded in insular mammals across different taxonomic and temporal scales [15–19]. This phenomenon is frequently accompanied by other anatomical modifications, including shortened limbs, bone fusions, modified dentition and/or reduced cranial pneumatization [15,20–23], indicating that insular dwarfs are not simply scaled-down versions of their mainland relatives.

Neuroanatomical evolution under phyletic insular dwarfism has received growing attention, especially regarding relative brain size changes [22,24–30]. A reduction in relative brain size has been observed in insular dwarf mammals, such as the Balearian mouse goat *Myotragus balearicus*, the Madagascan dwarf hippopotamuses *Hippopotamus madagascariensis* and *Hippopotamus lemerlei*, and the dwarf hominin of Flores *Homo floresiensis* [22,24,26,30,31]. However, the generality and magnitude of this pattern remain unclear, with other taxa exhibiting moderately smaller to larger brains compared to their closest mainland relatives [22,25,32]. Furthermore, different patterns of brain size reduction in island dwarfs have been recorded depending on the choice of the mainland reference sample. For instance, the relatively small brain of *M. balearicus*, approximately 17% smaller than that of Late Miocene bovids, may have been largely retained from its continental ancestor rather than being solely a result of island evolution [30].

**Figure 1.**
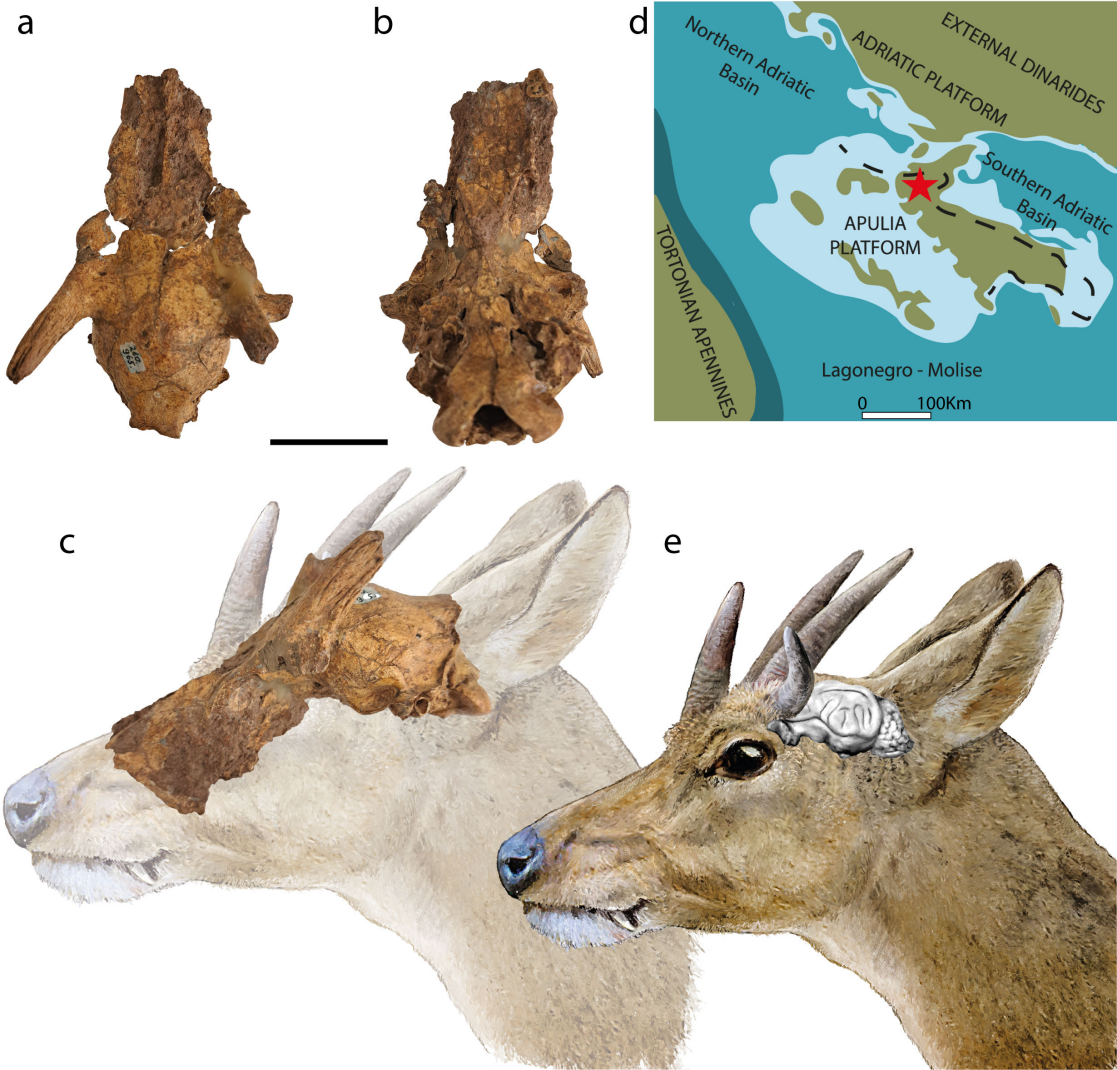
Skull and reconstruction of the Late Miocene ruminant *Hoplitomeryx matthei* from Gargano (Apulia, Italy). (a–c) External cranial morphology (holotype; RGM 260965): (a) dorsal, (b) ventral and (c) lateral views. Scale bars, 3 cm. (d) Palaeogeographic reconstruction of the Apulia Platform and adjacent platform-and-basin system during the Tortonian. Modified from Patacca *et al*. [33]. The red star indicates the position of Gargano, and the dashed line represents the present coastline of Apulia. (e) Life reconstruction of the head of *H. matthei*. Also shown are the relative positions of the skull and the brain (artist: Velizar Simeonovski).

The Late Miocene *Hoplitomeryx* is one of the oldest insular ruminants known thus far and inhabited the Gargano palaeo-island in Italy, a geographically isolated shallow-water domain during the Mesozoic and Cenozoic (figure 1) [33–35]. The unique traits that characterize this genus, such as the presence of five cranial appendages, make it difficult to reconstruct its biogeographical and evolutionary history [35]. Accordingly, its systematics and position within Ruminantia have been the subject of much debate since its discovery. Some authors placed *Hoplitomeryx* in the superfamily Cervoidea [34,35], while others have proposed a closer relationship to Bovidae [36–39], or a more basal position within Ruminantia [40,41]. The number of valid genera and species is also debated. One view proposes assigning the material to two genera: *Hoplitomeryx*, restricted to the Gargano palaeo-island, with four species, including the type species *H. matthei*; and *Scontromeryx*, from the older site of Scontrone (Abruzzo, Italy), with six species [35]. Mazza *et al.* [41], on the other hand, reject this split and recognize six species within *Hoplitomeryx* for both localities combined. Regardless of classification, the material points to a species radiation on the palaeo-island or palaeo-archipelago, with several *Hoplitomeryx* species co-occurring [42,43]. A key challenge in ascertaining the phylogenetic relationships of insular taxa lies in finding synapomorphic key characters, while distinguishing plesiomorphic features from homoplasies (secondary return to an ancestral condition or loss of a derived feature) driven by similar selective pressures of island environments [20,44,45]. *Hoplitomeryx* is no exception, as evidenced, for instance, by its non-parallel-sided astragali [20,23,46,47]. Although the shape of the astragalus has been used to suggest an ‘archaic origin’ of this genus [40,46], its overall postcranial morphology reflects the acquisition of ‘low gear’ locomotion, a peculiar type of gait frequently observed in island ruminants [15,20,23,47]. Little is known so far about the endocranial morphology of *Hoplitomeryx* and, more in general of insular mammals before the Quaternary, except for the brain size reduction reported for *Litovoi tholocephalos*, a multituberculate from the latest Cretaceous of Romania [28]. *H. matthei* is the oldest island ruminant for which the complete neurocranium is available and provides therefore a promising model to investigate brain evolution on islands in deep time.

Here, we use high-resolution X-ray computed tomography (CT), three-dimensional (3D) segmentation and modelling to obtain and describe the first digital endocasts of an insular ruminant, based on two skulls of *H. matthei*. We compare its brain morphology with that of 25 extant and 10 extinct relatives (Early Miocene to Holocene), including mainland and insular species. By integrating comparative morphology with an investigation of endocranial size and shape variation in our sample, we aim to shed light on the biogeographical history of *Hoplitomeryx* and on the antiquity of brain evolution patterns in insular mammals. In the context of the unique characteristics of *H. matthei*, the study of its endocast may prove relevant not only to contribute to our understanding of brain evolution in ruminants but also to help address broader questions of convergence and divergence in insular environments.

## 2. Material and methods

### (a) Institutional abbreviations

AMNH, American Museum of Natural History, New York, USA; FMNH, Field Museum of Natural History, Chicago, USA; RGM, Naturalis Biodiversity Center, Leiden, The Netherlands; ZMB, Museum für Naturkunde, Berlin, Germany.

### (b) Brain endocasts

We assembled a dataset of 35 neurocranial endocasts, including 27 digital endocasts based on high-resolution CT data and 8 physical endocasts (latex and natural). We obtained digital endocasts from two undistorted skulls of *H. matthei* housed at the Naturalis Biodiversity Center (Leiden, The Netherlands): the holotype (RGM 260965; figure 1) and an additional, fragmentary specimen (RGM 260944; electronic supplementary material, figure S1). Our comparative sample encompasses endocasts of 25 extant and 10 extinct ruminants (electronic supplementary materials, figures S3–S7). Notably, we segmented the first brain endocasts of the early bovid *Eotragus clavatus* and the early cervid *Euprox furcatus*, both dating to the Middle Miocene. The raw CT-scan data were exported as Tag Image File Format (TIFF) or DICOM stacks from CT laboratory computers at various institutions. Detailed information on all included individuals and scanning parameters for specimens scanned during this study is provided in the electronic supplementary material, tables S1 and S2. We manually segmented the endocranial cavity of each specimen with the software VGSTUDIO MAX (v. 3.4, Volume Graphics GmbH) using the tools ‘Region growing’, ‘Draw’ and ‘Smoothing’. The resulting 3D models were converted into surface meshes in PLY format [48]. These digital models were supplemented with latex and natural endocasts—all complete except for three—which were scanned using a Next Engine 3D laser scanner and converted into closed mesh models.

### (c) Morphological description and endocranial metrics

To describe the endocasts we relied on the anatomical nomenclature used in previous studies [49–52]. For each specimen, we measured total endocast volume (EV) and volume of the olfactory bulbs (ObV) in VGSTUDIO MAX. The olfactory bulbs were manually delineated along the circular fissure. The area of the exposed cortex was estimated in MeshLab^®^ v. 1.3.3 software [53] following Jerison [54], in which the neocortex is ventrally delimited by the rhinal fissure. To convert EV into brain mass, we applied the equation from Benoit [55]: brain mass = (0.8877 × EV) − 2.9408. The body mass of each individual, whose endocast was segmented in this study, was estimated using an equation developed for ungulate mammals based on the occipital condyle width (OCW): ln(body mass) = 7.6451 × ln(OCW)2/3 − 8.0565 [56]. We completed this dataset with brain and body mass estimates from the literature [25,30,57–59] electronic supplementary material, table S3).

We fitted ordinary least squares (OLS) and phylogenetic generalized least squares (PGLS) linear regressions to investigate the relationship between brain mass and body mass. We ran the OLS model to ensure comparability with previous studies and the PGLS model to account for phylogenetic dependence. The PGLS model was fitted using the ‘pgls’ function from the R package *caliper* (v. 1.0.3 [60]) with a phylogenetic topology based on multiple sources [39,61,62] (electronic supplementary material, text and figure S8).

To assess differences in relative brain size between *H. matthei* and ruminant families, we computed the encephalization quotient (EQ) using an equation based on a sample comprising only artiodactyls, while excluding cetaceans [63]. We also calculated the relative area of the exposed cortex (RAEC) as the ratio between the measured surface area and EV to the power of two-thirds. We used RAEC to quantify the relative size of the neocortex [64]. We performed Fisher–Pitman permutation tests to compare EQ, RAEC and EV and ObV (electronic supplementary material, table S4). This non-parametric test was selected due to unequal sample sizes, with some groups containing only a single observation [65]. Additionally, we conducted pairwise permutation tests to assess differences in EQ between *H. matthei* and ruminant families, using 1000 permutations per comparison (electronic supplementary material, table S5). To control for false positives, we adjusted *p*-values for multiple comparisons using the ‘fds’ method [66].

### (d) Geometric morphometrics

We generated a sample to examine endocast shape variation across Pecora, with emphasis on the relative position of *H. matthei* within this clade (electronic supplementary material, table S6) [67]. We adapted protocols included in Bertrand *et al.* [68], Fontoura *et al.* [57] and Lang *et al.* [69] and used Landmark Editor [70] to place 14 fixed landmarks and one curve consisting of 10 semilandmarks on the right hemisphere of each endocast (electronic supplementary material, figure S9). All statistical and geometric morphometric analyses were conducted in R (v. 4.2.1 [71]). Geometric morphometrics analyses were conducted using the package *geomorph* (v. 4.2.3 [72]). To include four key fossil taxa for which no complete endocast was available to us in our analyses, we imputed missing landmarks using the thin-plate spline interpolation method via the ‘estimate.missing’ function (electronic supplementary material, figure S9). We then conducted a generalized Procrustes analysis to rotate, translate and scale the raw landmark configurations, resulting in Procrustes shape coordinates [73]. For each endocast, we obtained a size proxy, that is, the centroid size (i.e. the square root of the sum of squared distances between landmarks and their centroid), and Procrustes coordinates which represent its shape [73]. We calculated mean shapes for species represented by more than one individual. GPA was performed using the ‘gpagen’ function.

To assess shape variation and evaluate the morphological affinities of *H. matthei*’s endocast within our sample, we performed a principal component analysis (function ‘gm.prcomp’). Shape changes along principal component axes were visualized using the function ‘plotRefToTarget’ (electronic supplementary material, figure S10). Furthermore, we projected our pruned phylogenetic tree into the morphospace defined by the first four principal components using the ‘phylomorphospace’ function from the *phytools* package (electronic supplementary material, figure S11). To evaluate the effect of allometry we tested for a correlation between centroid size and Procrustes coordinates using a phylogenetic ANOVA and a pruned version of the composite phylogenetic tree (function ‘procD.pgls’; electronic supplementary material, table S4). Finally, we tested for phylogenetic signal in Procrustes shape coordinates by running the function ‘phylosig’ in *phytools* [74], which calculates Blomberg’s *K* and Pagel’s *λ*.

## 3. Results

### (a) Description and comparison

**Figure 2.**
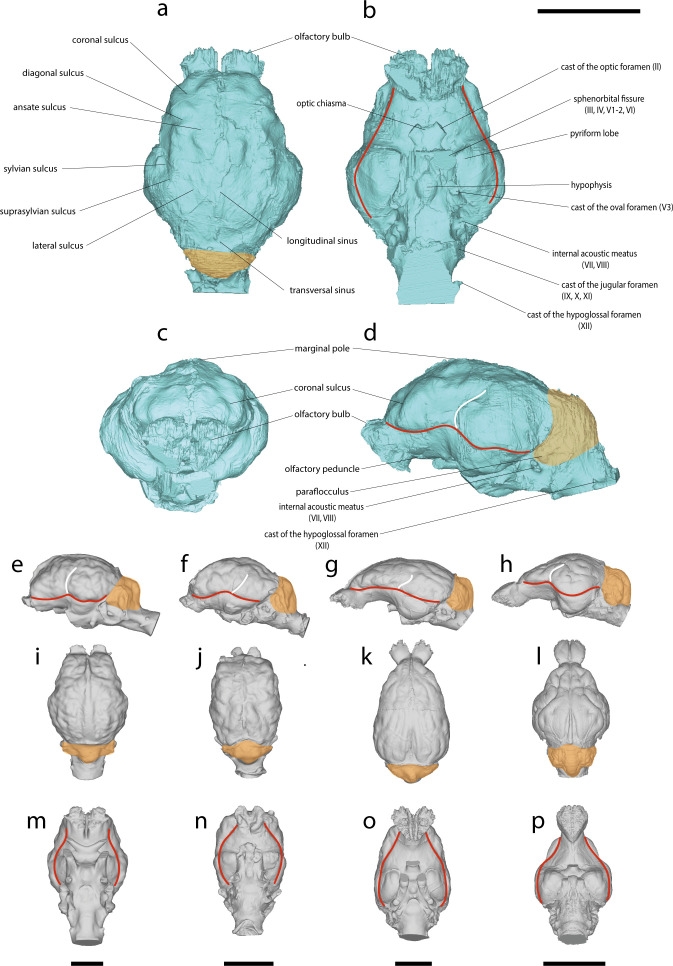
Brain endocasts of *Hoplitomeryx matthei* (RGM 260965) (a–d), *Capra sibirica* (AMNH 54906) (e,i,m), *Myotragus balearicus* (FMNH PM 59151) (f,j,n), *Muntiacus muntjak* (ZMB MAM 40556) (g,k,o) and *Tragulus napu* (ZMB MAM 66594) (h,l,p) in dorsal (a,i–l), ventral (b,m–p), lateral (d,g,k) and anterior (c) views. Coloured area and lines indicate: orange, cerebellum; red, rhinal fissure; white, sylvian sulcus. Roman numeral designations indicate cranial nerves. Scale bars, 3 cm.

*Hoplitomeryx matthei* has a gyrencephalic brain endocast, with well-separated olfactory bulbs that appear triangular in ventral view, and short olfactory peduncles (figure 2). The pyriform lobe forms a prominent, anteroventrally oriented projection on the endocast (figure 2). The rhinal fissure delimits the neocortex and is positioned at approximately 25% of the brain’s total height measured from the ventral surface. In lateral view, the rhinal fissure exhibits an angle between its anterior and posterior parts, with the transition between them forming an arc (figure 2). The sylvian (or pseudo-sylvian) sulcus is slightly convex. The suprasylvian sulcus forms an arch over the sylvian gyrus. The posterior part of the sylvian gyrus is broader than its anterior part. The coronal sulcus measures approximately one-third of the total length of the cerebral cortex (figure 2; electronic supplementary material, figures S1 and S2). The diagonal sulcus extends dorsolaterally, beginning near the coronal sulcus and running towards the rhinal fissure (figure 2; electronic supplementary material, figures S1 and S2). The ansate sulcus, located at the limit between the frontal and the parietal lobes, marks the anterior boundaries of a pronounced marginal pole, the highest anatomical feature of the endocast. There are traces of oblique sulci (electronic supplementary material, figure S2). On the caudal portion of the dorsal surface, there is a lateral sulcus that runs almost parallel to the longitudinal fissure. Anteriorly, it is slightly more medial than posteriorly. There is no ectolateral sulcus. The midbrain is entirely overlain by the neocortex (figure 2; electronic supplementary material, figures S1 and S2). The cerebellum has a poorly defined vermis and poorly developed lateral hemispheres, but the paraflocculi can still be observed (figure 2). On the ventral surface, the impression of the optic foramen is only preserved in RGM 260965 and is relatively wide (figure 2). The dome-shaped hypophysis is well defined and positioned along the midline of the ventral aspect of the endocast, between the impressions of the sphenorbital fissure and the oval foramen. Posterior to these, the internal acoustic meatus is visible, as well as the impressions of cranial nerves, including the hypoglossal and jugular nerves (figure 2).

The brain of *H. matthei* exhibits a combination of characters also observed in Pecora (figure 2; electronic supplementary material, figures S1–S7 and S12). Contrary to what is observed in tragulids, olfactory bulbs are well separated and similar in shape to those of *Capra sibirica* and *Moschus moschiferus*. The frontal lobe of *H. matthei* is more developed than in tragulids (figure 2; electronic supplementary material, figure S5). When compared to *Muntiacus muntjak* and *Tragulus napu*, which possess more elongated peduncles, those of *H. matthei* are notably shorter and more robust, a characteristic more consistent with *Saiga tatarica*, members of the Caprini tribe, and Antilocapridae. The constriction at the sylvian sulcus visible in dorsal view in *H. matthei* is absent in cervids and *Cephalophus leucogaster*. Additionally, the developed marginal pole exhibited by *H. matthei* can only be observed in some Caprini in our reference sample, such as *C. sibirica* and *Oreamnos americanus. H. matthei* displays a sulcal pattern resembling *E. furcatus*, *E. clavatus* and *M. moschiferus*. Its cerebellum is similar to *Rupicapra rupicapra* and *Urmiatherium intermedium*, as it is only slightly extended dorsally and has a poorly defined vermis. The hypoglossal and jugular nerves exhibit a configuration similar to that seen in Caprini (figure 2; electronic supplementary material, figure S3). Finally, the dome-shaped hypophysis of *H. matthei* resembles those of *C. sibirica*, *E. furcatus* and Antilocapridae. Detailed descriptions and comparisons can be found in the electronic supplementary material.

### (b) Encephalization and brain component percentage comparisons

**Figure 3.**
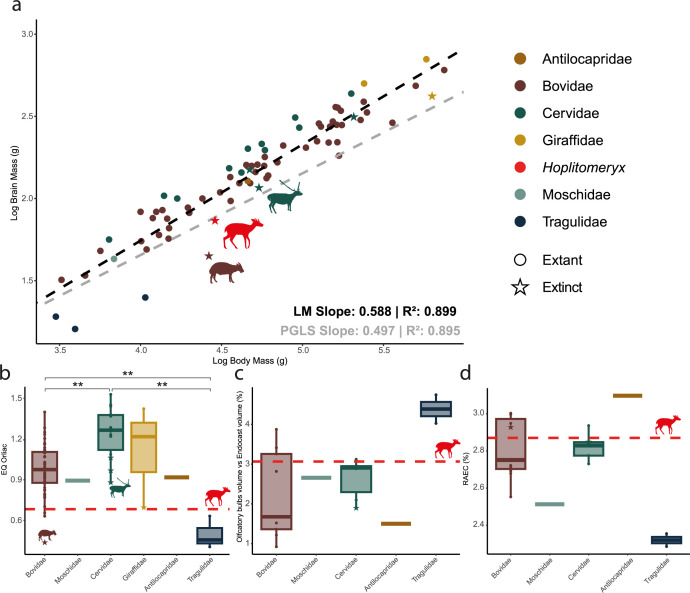
Comparisons of endocranial metrics in ruminants. (a) OLS and PGLS regressions of log-transformed values of brain and body mass. Boxplots of (b) EQ based on the equation from Orliac *et al.* [63], (c) olfactory bulbs volume percentage and (d) RAEC in our sample. Dashed red lines indicate estimated values for *Hoplitomeryx matthei* (**families with significantly different EQ based on pairwise permutation tests; electronic supplementary material, table S5). Silhouettes represent insular endemic species: *H. matthei*, *Myotragus balearicus* and *Candiacervus ropalophorus*.

*Hoplitomeryx matthei* has a brain volume (EV) of 85 972.52 mm^3^ and a brain mass of 73.38 g, based on the specimen RGM 260965. We estimated the body mass of this individual to be 28.82 kg. Log-transformed brain and body mass estimates for *H. matthei* and our comparative sample are displayed in figure 3a. Both OLS and PGLS models show a highly significant correlation between brain and body mass (figure 3a; electronic supplementary material, table S4). Like the insular deer *Candiacervus ropalophorus*, *H. matthei* falls below the OLS regression line, indicating that its brain mass is lower than expected for a ruminant of the same body mass. However, *H. matthei*’s brain mass reduction is less pronounced than that of *M. balearicus,* and its brain is relatively larger than that of Tragulidae.

We obtained an encephalization quotient (EQ) value of 0.68 for *H. matthei* based on specimen RGM 260965. This value is higher than those of Tragulidae and *M. balearicus* (EQ = 0.44), but lower than most of the Pecora taxa in our sample. Some Pecora, like extant Caprini, such as *O. ammon* (EQ = 0.63), exhibit values close to that of *H. matthei* (figure 3b; electronic supplementary material). Results of Fisher–Pitman permutation tests indicate a significant variation in EQ among ruminant families (*p* < 0.001; electronic supplementary material, table S4). In particular, pairwise comparisons of EQ values show significant differences between Bovidae and Cervidae, Bovidae and Tragulidae, and Cervidae and Tragulidae (figure 3b; electronic supplementary material, table S5). However, there are no significant differences between the EQ of *H. matthei* and those of most families (Bovidae, Giraffidae, Moschidae and Tragulidae). Only Cervidae show significantly different EQ values compared to *H. matthei* (*p* = 0.02), although this difference becomes non-significant after adjusting for multiple comparisons (*p* = 0.10).

The olfactory bulbs of RGM 260965 represent 3.07% of its total brain volume (figure 3c; electronic supplementary material, table S1); thus they do not appear particularly reduced. Tragulids are characterized by the highest ObV in our sample, with *Moschiola meminna* showing an ObV percentage of 4.03% and *T. napu* of 4.74%. Among Pecora, *C. leucogaster* and *C. silvicultor* exhibit relatively high ObV ratios (3.87% and 3.41%, respectively), while species like *O. americanus* show lower ratios (0.92%). The difference in ObV percentage between Tragulidae and the other families is only apparent (figure 3c), as we found no overall support for a significant variation among the ruminant families in our sample (*p* = 0.13; electronic supplementary material, table S4).

The RAEC of RGM 260965 is 2.87. In our sample, the values range from 3.10 (*Antilocapra americana*) to 2.29 (*T. napu*). The boxplots and the Fisher–Pitman permutation test show significant differences in RAEC among the ruminant families in our sample (*p* = 0.02; figure 3d; electronic supplementary material, table S4). Tragulids are distinct from Pecora, and the value for *H. matthei* aligns with those observed in Pecora (figure 3d).

### (c) Principal component analysis

**Figure 4.**
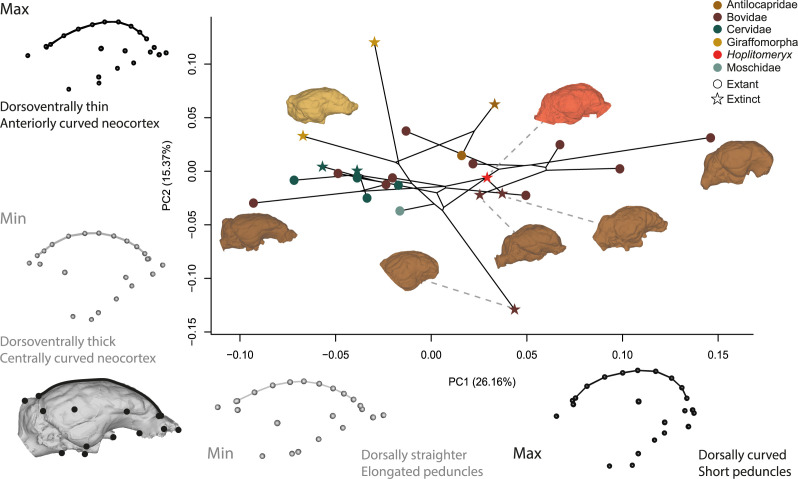
Phylomorphospace of the first two principal components (PC1 versus PC2) based on each species’ 3D Procrustes shape coordinates. Models (lateral view) show minimum (Min) and maximum (Max) shape variation along PCs. Endocasts from left to right: *Cephalophus leucogaster*, *Prolibytherium magnirei*, *Eotragus clavatus*, *Myotragus balearicus*, *Hoplitomeryx matthei*, *Urmiatherium intermedium* and *Rupicapra rupicapra*. Dorsal views, models of PC3 versus PC4 and landmarking protocol can be found in electronic supplementary material, figures S9–S11.

The first two principal components together explain 41.26% of the total shape variation in our sample, with PC1 representing 25.6% of the variation. Minimum PC1 values correspond to elongated, dorsally flattened endocasts with anteriorly developed and ventrally oriented olfactory bulbs (figure 4). Maximum PC1 values reflect globose endocasts, characterized by a more prominent neocortex and reduced dorsal extension of the cerebellum. Furthermore, the olfactory bulbs are shorter and the lateral-most point of the endocast is more anteriorly projected. Minimum PC2 values correspond to endocasts that are dorsoventrally developed. In lateral view, the neocortex exhibits its most pronounced curvature at the centre. Additionally, the lateral-most point of the endocast in the dorsal view is projected posteriorly and the cerebellum is short (figure 4b; electronic supplementary material, figure S10). Maximum PC2 values are associated with dorsoventrally thinner endocasts, where the curvature of the longitudinal sulcus is most pronounced in the anterior region of the neocortex. The cerebellum is also posteriorly more developed. The eigenvalues, the variance explained by all the components and the scatterplot of PC3 versus PC4 can be found in electronic supplementary material, figures S10 and S11 and table S7.

In the morphospace delimited by PC1 and PC2, *H. matthei* is positioned in the positive values of PC1, closer to the Miocene bovid *U. intermedium* and the insular Caprini *M. balearicus*. Bovidae occupy the broadest region (figure 4), with *C. leucogaster* having the lowest PC1 score, *R. rupicapra* the highest PC1 score and *E. clavatus* with the lowest PC2 score. In contrast, both extant and extinct Cervidae in our sample occupy a more restricted region of the morphospace with low values for both PC1 and PC2, and overlap with the Moschidae *M. moschiferus*. Giraffomorpha have negative PC1 values and positive PC2 values, with *S. boissieri* at the maximum extreme of this distribution. Antilocapridae have positive values for both PC1 and PC2, close to zero.

### (d) Endocranial shape allometry and phylogenetic signal

Results of phylogenetic ANOVA show no significant relationship between centroid size and shape (*p* = 0.29; electronic supplementary material, table S4). Both Pagel’s *λ* (0.97, *p* = 0.03) and Blomberg’s *K* (0.71, *p* = 0.04) indicate the presence of a significant phylogenetic signal in our sample (electronic supplementary material, table S4). Pagel’s *λ* is close to 1, suggesting that endocast shape has largely evolved according to Brownian motion. Blomberg’s *K* indicates a moderate signal, suggesting that while phylogeny plays an important role, other factors may also contribute to brain endocast shape variation.

## 4. Discussion and conclusion

The brain endocasts of *H. matthei* provide new insights into its phylogenetic affinities and biogeographical history. Several neuroanatomical features shared by *H. matthei* and pecoran ruminants, such as well-separated olfactory bulbs, and a relatively expanded frontal lobe, support the exclusion of this taxon from Tragulina (figures 2 and 3; electronic supplementary material, figure S12) [63]. Furthermore, the EQ and RAEC values of *H. matthei* are higher than those estimated for the tragulids in our sample (figure 3) and for the Oligocene ruminants reported by Orliac *et al.* [63]. Notably, the rhinencephalon and neocortex external shape in *H. matthei* closely resembles that of Bovidae (figures 2 and 4; electronic supplementary material, figure S3). The presence of a well-developed marginal pole reported only in Bovini in ruminants outside Caprini further reinforces this affinity (figure 2) [49,75]. These traits indicate that *H. matthei* had a relatively derived pecoran brain and support a closer phylogenetic relationship with Bovidae than with other ruminant families, as also indicated by the morphology of its inner ear and presence of horn-cores [36–39]. Based on the different hypotheses regarding the phylogenetic position of *Hoplitomeryx* within Ruminantia, two colonization scenarios for Gargano have been proposed. The first suggests a late Oligocene dispersal via land bridges from the Balkans, with *Hoplitomeryx* deriving from a small-sized Oligocene Tragulina [46,76,77]. The alternative hypothesis proposes that the ancestor of *Hoplitomeryx* was a Miocene member of Pecora, which would have colonized Gargano via an overseas sweepstake dispersal route [35,78,79]. The derived pecoran endocranial morphology of *H. matthei* aligns with the latter scenario and suggests that the colonization of Gargano by its putative ancestor likely occurred no earlier than the Early Miocene.

Various degrees of brain size reduction have been reported in insular dwarfed mammals [22,25,32]. A moderate reduction in brain size appears to be the most common pattern and may be linked to the brain and sense organs reaching their maximal size before the rest of the body during ontogeny [26]. *H. matthei* has an EQ lower than that of the smallest Cretan deer *C. ropalophorus*, but higher than that recorded for *M. balearicus* (figure 3) [24,25,30]. The magnitude of brain size reduction in insular dwarf species largely depends on the choice of its putative mainland ancestor [22,30]. In this regard, if *H. matthei* is related to bovids, as its endocranial morphology suggests, then its EQ falls within the family’s range of variation (figure 3), indicating that brain size reduction, if present, was moderate. On the other hand, a more marked, yet not extreme, brain size reduction could be inferred if an affinity with other pecoran families is confirmed. The absence of a strongly reduced brain size in *H. matthei* may result at least in part from the lack of reduction of its occipital cortex and olfactory bulbs, which is instead well documented in *M. balearicus* [24,30]. The differences in brain size and morphology between these two insular ruminants may reflect distinct selective pressures on their respective islands. The pronounced ecological release from competition, predation and resource limitation experienced by *Myotragus* on the Balearic islands may have driven a greater reduction in brain size and complexity [24,25,30]. Conversely, the fauna of Gargano was more balanced and less impoverished, with the presence of intra-generic competitors and potential predators (the giant eagle *Garganoaetus*, giant barn owl *Tyto gigantea* and the crocodile *Crocodylus* sp.), which may have exerted strong selective pressures on *H. matthei*’s brain and its sensory functions [42,43,80]. Similar pathways in brain evolution may be invoked for the smallest *Candiacervus* species from Crete, whose brain exhibits less pronounced modifications than *M. balearicus* [25], and for the Sardinian Late Pliocene–Early Pleistocene endemic bovid *Nesogoral melonii*, which shares neuroanatomical traits with *H. matthei*, such as a relatively well-developed frontal lobe and a prominent marginal pole [30,81]. Rather than exhibiting the striking modifications seen in *M. balearicus*, *C. ropalophorus*, *N. melonii* and *H. matthei* show less marked neuroanatomical changes, which may be explained in light of inter- and intra-generic competition dynamics, leading to species radiations within these monophyletic lineages [43,45,82–84]. Ecological displacement and evolutionary diversification in these taxa—likely reflecting greater habitat complexity on Crete, Sardinia and Gargano compared to the Balearic Islands [23–25,33]—might have prevented extreme brain modification in their dwarfed representatives.

Taken together, these findings attest to the antiquity of changes in brain size and shape in island ruminants before the Quaternary, while highlighting the substantial variability in the magnitude of these patterns. Neuroanatomical changes in insular phyletic dwarfs appear to be shaped by a combination of phylogenetic constraints and island-specific selective factors. This hypothesis, albeit largely untested, aligns with the growing recognition that morphological evolution on islands results from the complex interplay of contextual biotic and abiotic parameters, reflecting a variety of ecological mechanisms.

## Data Availability

The article's supporting data can be found in the electronic supplementary material. The surface renderings of the endocasts are deposited on the repository Morphosource at Hoplitomeryx dataset // MorphoSource. Access to the 3D models will be approved by P.O. and R.R. The endocasts cannot be described without permission from P.O. and R.R. The brain endocast of *Antifer ensenadensis* described by Fontoura *et al.* is available on MorphoMuseuM [85]. The code used for this study is available at Zenodo [67]. Supplementary material is available online [86].
